# Dr. Ferguson on the Prevention of Typhus
†This was originally addressed to a gentleman in Aberdeen.


**Published:** 1818-07

**Authors:** 


*For the London Medical and Physical Journal.*

### Dr. Ferguson *on the Prevention of Typhus.*†

OF all the diseases which affect the human species, Fever appears to be the most frequent, and the most uncertain in its cure. When epidemic, it generally puts on the nature of Typhus, and affects the nervous system violently and fatally: the progress of which no medicine can arrest. It is of great consequence to prevent the infection of fever, and many different methods have been tried, some of which are no doubt very powerful. The nitrous vapour of Dr. Smith, and the muriatic, are the most to be depended on for this purpose. These are prepared by putting either nitre or common salt into a cup, and adding sulphuric acid as long as the vapour continues to rise. This, with free ventilation,

† This was originally addressed to a gentleman in Aberdeen.

will have a great effect in destroying the infection in chambers where the sick are confined; and when this is punctually observed, the assistants and physician will require no other preventative to guard against its effects. Snuff and camphor have been used for preventing infection; and, in my Essay on Fever, you will find these observations:—P. 189, "Stimulating the olfactory nerves, by fragrant essential oils, is also a means of wearing out the accumulation of the vitality, and is a practice not often pursued in fever; yet, when we perceive the great connexion between the brain and the nerves of the nose, we must be convinced that a gradual and pleasant stimulus may be afforded to the nervous system by these means, while, at the same time, substances may be employed that will have a very great effect in destroying contagion." A little cotton dipped in a mixture of concentrated vinegar and camphor will have more influence in resisting contagion than snuff; but, if this cannot be obtained, snuff perfumed with the essential oil of peppermint, or dissolved camphor, may be had recourse to with advantage. Smoking tobacco is often used in places where intermittents are endemic, with the view of resisting the noxious power of the marsh-miasmata, which is the remote cause of this species of fever: but it may be equally serviceable in typhus, although it originates from a different contagion.

I would recommend to those who are much exposed to infection to bathe such parts of the body as are liable to its action with good vinegar, and, at the same time, to take a glass of wine before visiting patients, as nothing gives a greater power to contagion than the depressing passions, which are among the number of the remote causes of fever. Yeast has been long used in fever, and its good effects depend upon the carbonic acid, or fixed air, which it contains. The saline mixture, or acetate of ammonia, has been long used in fever, and its good effects must, in a great measure, depend upon the carbonic acid, or fixed air, which it contains; it ought, therefore, to be given in a state of effervescence. Yeast has been employed by different practitioners with the same view; and, as it is cheaper, it has been more frequently used among the lower classes; but, from the great quantity of glutinous matter which it contains, is often very disagreeable to the patients' stomachs. I recommend the carbonic acid to be swallowed, as it is emitted from the mouth of a bottle of brisk beer, which I think is a more effectual and grateful mode of administering the carbonic acid gas.—See p. 222, of my Essay on Fever. As this gas is found of so much use in typhus, I think the Seltzer water, which contains a great quantity of this gas, would be a very good mode of giving it, and very agreeable to the patients' stomachs; especially, as they are desirous of any medicine in the aqueous state. I have tried it, and the patients were very fond it, when all other medicines were rejected.

---

*For the London Medical and Physical Journal.*

*Cases of Hysteria from Indigestion;* by B., M. D.

